# Supplementary material for: Shifts in flood generation processes exacerbate regional flood anomalies in Europe
Source: Commun Earth Environ. 2023 Feb 23;4(1):49. doi: 10.1038/s43247-023-00714-8 (PMC11041756; doi:10.1038/s43247-023-00714-8)
Supplement: Supplementary file 2 — Supplementary Information [file 43247_2023_714_MOESM2_ESM.pdf]

Shifts in flood generation processes exacerbate regional flood anomalies in Europe

Larisa Tarasova[1], David Lun[2], Ralf Merz[1,3], Günter Blöschl[2], Stefano Basso[1,4], Miriam Bertola[2], Arianna Miniussi[1], Oldrich Rakovec[5,6], Luis Samaniego[5], Stephan Thober[5], Rohini Kumar[5]

[1] Department Catchment Hydrology, Helmholtz Centre for Environmental Research – UFZ, Halle (Saale), Germany

[2] Institute of Hydraulic Engineering and Water Resources Management, Vienna University of Technology, Vienna, Austria

[3] Institute of Geosciences and Geography, Martin-Luther University Halle-Wittenberg, Halle (Saale), Germany

[4] Norwegian Institute for Water Research (NIVA), Oslo, Norway

[5] Department Computational Hydrosystems, Helmholtz Centre for Environmental Research – UFZ, Leipzig, Germany

[6] Faculty of Environmental Sciences, Czech University of Life Sciences Prague, Prague-Suchdol, Czech Republic

Supplementary Information

Supplementary Note 1. Linking catchments to the model grid

We link catchments from the European Flood Database to the 5 km mHM grid using outlet coordinates and provided area of catchments. We were able to link 1444 (61% of all available catchments, Supplementary Figure 1) with the tolerance of 50% in the absolute area error. Adequacy of this threshold is examined with regard to model performance (see Supplementary Note 3). 34% of catchments that are not linked to the mHM grid are located outside of the model domain in the Eastern part of Europe (Russia, Ukraine and Turkey; Supplementary Figure 1).

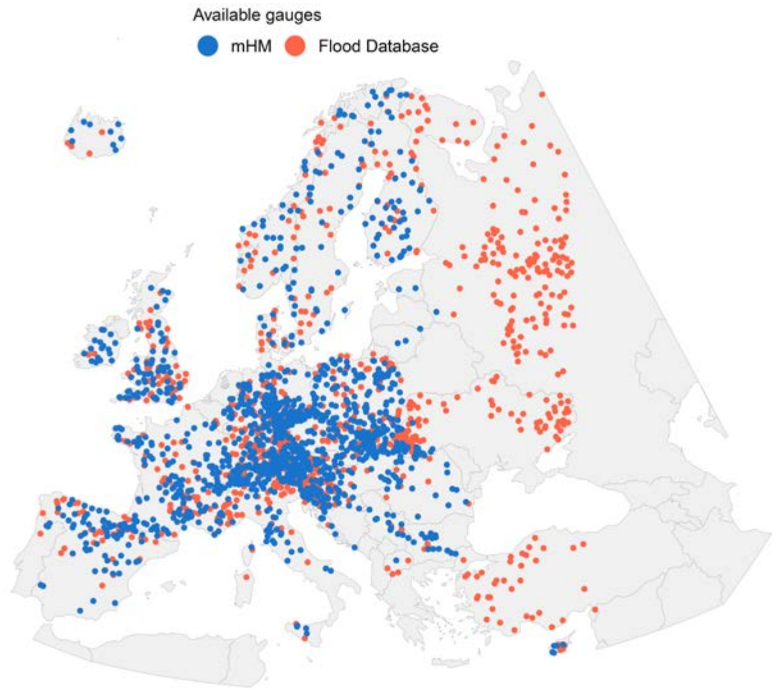

Supplementary Figure 1. Location of the gauges available from the European Flood Database and gauges that were linked to the mHM model grid.

The median size of the linked catchments is 349 km^2. The area distributions of catchments in the original European Flood Database and catchments linked to the mHM grid is almost similar (Supplementary Figure 2).

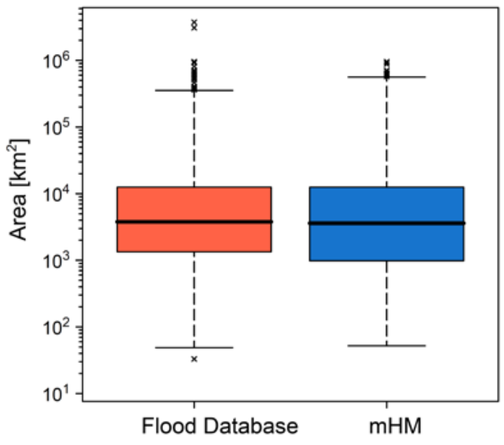

Supplementary Figure 2. Area of available catchments in the European Flood Database and in the subset of them that were linked to the mHM grid. Centre line of the boxplot corresponds to the median, upper and lower box corresponds to the interquartile range, the length of the whiskers is defined as 1.5 times the interquartile range, the outliers outside of this range are indicated as crosses.

Among these 1444 linked catchments 1353 have long enough time series (see Methods in the Main manuscript) for identification of flood anomalies and were finally selected for the analysis performed in this study.

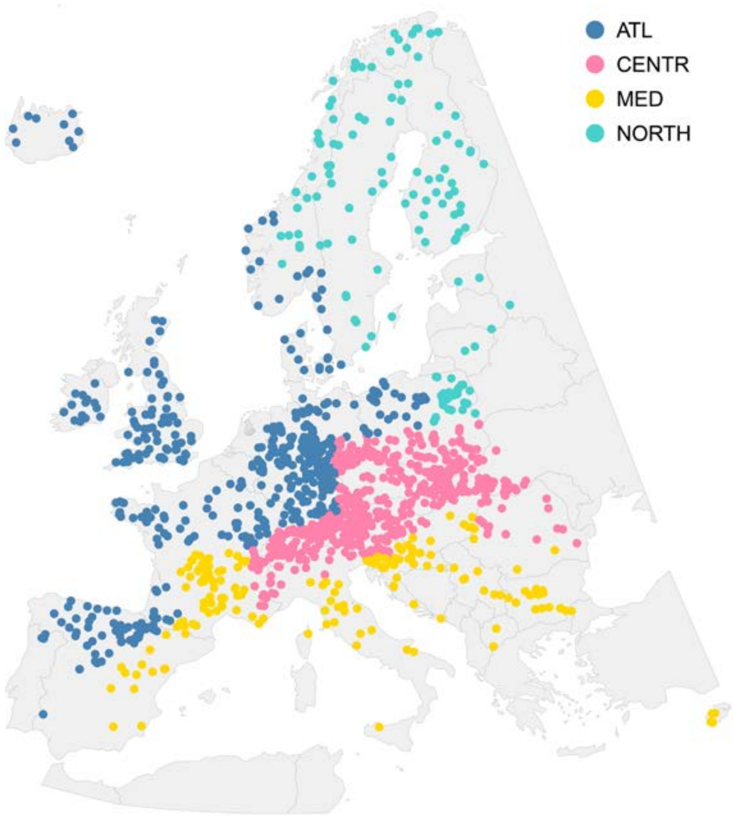

Supplementary Figure 3. Location of the study catchments organized into four regions in Europe. The Northern region (NORTH) consists of 124 catchments; the Atlantic region (ATL) includes 477 catchments; the Mediterranean region (MED) has 182 catchments and the Central-Alpine region (CENTR) has the remaining 570 catchments. Regions are defined similarly to Lun et al.[3]

Supplementary Note 2. Identification of flood events

Observed rainfall and simulated streamflow and snowmelt time series are used to identify runoff events using an automated time-series-based event separation[1]. The original method for event identification was developed for mesoscale German catchments and consists of three steps: baseflow separation using simple smoothing method[2], rainfall and snowmelt attribution using seasonal median lag time[3] and an iterative procedure for refinement of multiple-peak events driven by comparing distributions of event runoff coefficients (ratio of event quickflow volume and volume of event precipitation) of single-peak and multiple-peak events. When applying this method to a much more diverse set of European catchments an issue with exceedingly steep baseflow increases occurred in three cases: seasonal increase of baseflow in snow-dominated catchments[4]; long multiple-peak events in large catchments; multiple-peak events in rainfall-dominated catchments with very high frequency of rainfall events. Accordingly, we identify catchments where these issues might arise as snow-dominated catchments with snow contribution of at least 15% of total precipitation[5] (175 catchments), catchment with area larger than 30,000 km^2 (32 catchments) and to rainfall-dominated catchments where portion of dry spells is less than 50% of total time series duration (52 catchments). For these catchments we identify events with the slope of baseflow increase higher than 75th percentile of all events and merge them with the following event. This increases the length of the corrected events and consequently decreases the slope of baseflow increase. The procedure is repeated until all slopes exceeding the specified threshold are corrected.

Supplementary Note 3. Performance of the hydrological model: timing and magnitude of annual flood events

We evaluate model performance in terms of its ability to simulate observed peak discharges and timing of maximum annual floods to assure that the simulations are suitable for extracting the beginning and end points of flood events required for classification of flood events. Three different cases are considered. In the first case we compare observed and simulated maximum annual floods (*MAF definition*) in terms of their discharge and day of occurrence. In the second case we compare observed and simulated discharge on the exact date of the observed annual floods (*MAF date*). Finally, in the third case we compare observe discharge and date of maximum annual flood to the peak of corresponding runoff event (*MAF event*, Supplementary Figure 4).

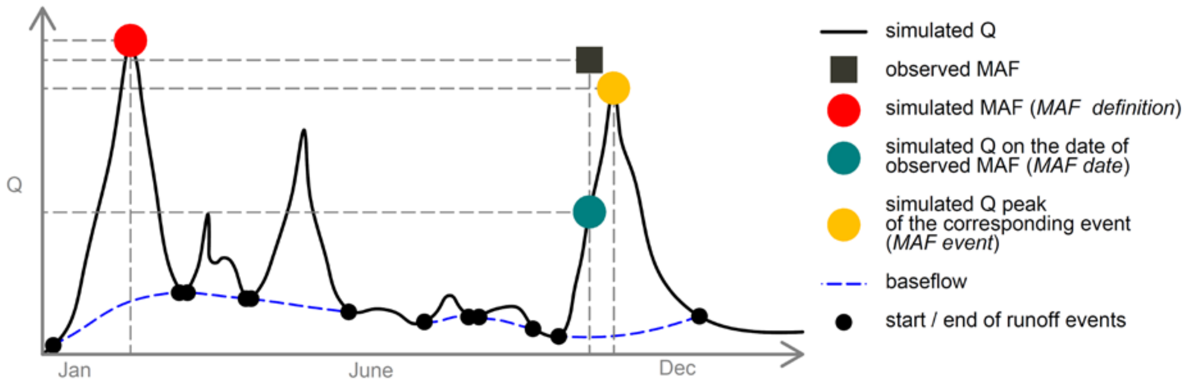

Supplementary Figure 4. Characteristic points for comparison of simulated and observed maximum annual floods. MAF date is the date of the observed annual maxima according to the European Flood Database. MAF definition is the date of annual maxima according to the mHM simulations. MAF event is the peak discharge of the runoff event that corresponds to the date when the observed annual maxima has occurred. In case of the perfect performance these three dates are identical. Timing errors are computed as the differences between these dates. Magnitude errors are computed as differences in peak discharges between these dates and the observed peak discharge of the corresponding annual maxima (see Supplementary Figure 5).

Spearman rank correlation r [-] is used to evaluate model performance in terms of flood magnitudes. The performance in terms of timing is evaluated by the absolute difference in [days] between the date of the observed annual flood and the date of the simulated annual flood/corresponding runoff event.

The model shows adequate performance in terms of the magnitudes of simulated peak discharges of annual floods: the mean Spearman rank correlation r [-] is 0.53-0.60 for all study catchments for three different comparison cases (Supplementary Figure 5a). Although the differences of the dates of the simulated and observed annual floods are sometimes substantial (Supplementary Figure 5b, MAF definition), the model is able to simulate corresponding runoff event of comparable magnitude with minimal timing errors in the absolute majority of cases (Supplementary Figure 5b, MAF event and Supplementary Figure 5c) indicating that the mHM simulations are suitable for further analysis of flood generation processes across European catchments.

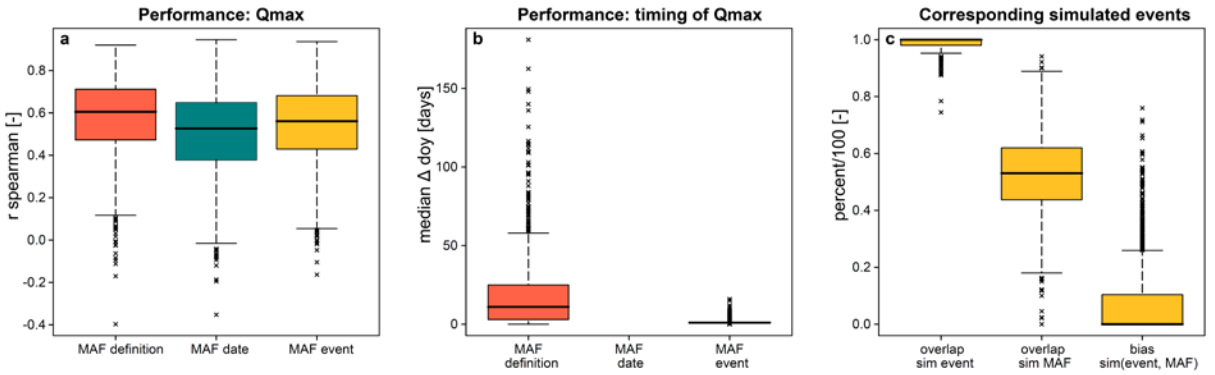

Supplementary Figure 5. Model performance in terms of timing and magnitude of annual flood events. **a** Spearman rank correlation r [-] between observed peak discharges of annual maximum floods and simulated peak discharges for three different cases: MAF date is the date of the observed annual maxima according to the European Flood Database; MAF definition is the date of annual maxima according to the mHM simulations; MAF event is the peak discharge of the runoff event that corresponds to the date when the observed annual maxima has occurred. **b** Median difference in the day of occurrence (doy) observed maximum annual floods and corresponding model simulations for three different cases. **c** Comparison of the observed annual floods with the corresponding simulated runoff event: portion of cases when the observed maximum annual flood was recorded during a simulated runoff event; portion of cases when the observed maximum annual floods was recorded during a simulated runoff event that has a highest annual peak discharge (i.e., also a maximum annual flood according the model simulations); Percent difference between the simulated peak discharge of the runoff events that corresponds to the observed maximum annual flood and the simulated peak discharge that corresponds to the maximum annual flood according to the model definition (i.e, differences in the magnitudes of MAF event and MAF definition, Supplementary Figure 4). Centre line of the boxplot corresponds to the median, upper and lower box corresponds to the interquartile range, the length of the whiskers is defined as 1.5 times the interquartile range, the outliers outside of this range are indicated as crosses.

Additionally we examine the role of discrepancies in the catchment area that occurred from linking catchments to the 5 km mHM grid (see Supplementary Note 1). Supplementary Figure 6 shows that the selected tolerance margins (i.e., maximum 50% of area discrepancy) is suitable for the purpose of this study as there is no visible deterioration of model performance with increasing area discrepancy both in terms of timing and magnitude.

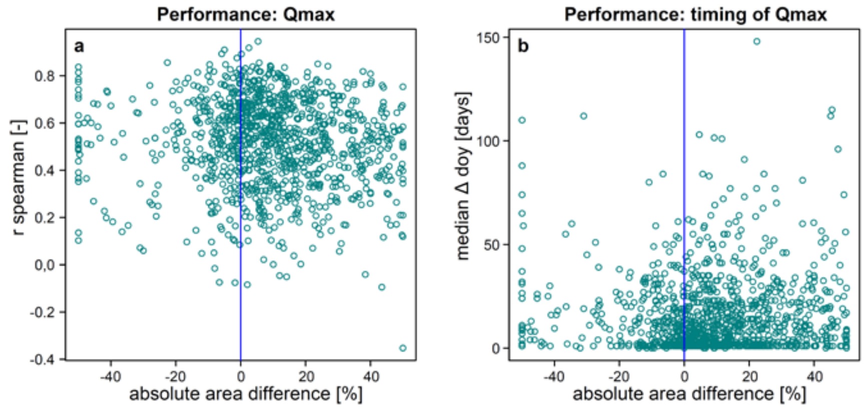

Supplementary Figure 6. Model performance as function of catchment area discrepancy. **a** Mean Spearman rank correlation r [-] between observed peak discharges of annual maximum floods and simulated peak discharges for MAF date case (see Supplementary Figure 4). **b** Median difference in the day of occurrence (doy) observed maximum annual floods and corresponding model simulations for MAF date case (see Supplementary Figure 4).

Supplementary Note 4. Causative classification of flood events

Each flood event is attributed to one of the four event types according to the process-based framework for event characterization and classification[6]: 1) Rain.Dry (flood events generated by rainfall on dry soils), 2) Rain.Wet (flood events generated by rainfall on wet soils), 3) Rain.Snow (flood events generated by the mixture of rainfall and snowmelt), and 4) Snowmelt (flood events generated by snowmelt). Two indicators are used for this classification: The first indicator is the portion of catchment- and event-averaged snowmelt ($M_{x,y,t}$) relative to the total volume of precipitation ($P_{x,y,t}$) event. The second is the catchment-averaged antecedent soil moisture one day prior the start of the

corresponding flood event ($SM_{x,y}(t_0)$). For both indicators critical thresholds are selected (Supplementary Figure 7).

For the antecedent soil moisture max(κ) is selected as the critical threshold that signifies the point of maximum curvature of the fitted exponential function that describes a non-linear behaviour of runoff event coefficients and antecedent soil moisture for all runoff events identified by the automated event separation procedure[1]. Below this threshold, event runoff coefficients are rather low and increase only slightly with increasing soil moisture. This corresponds to dry antecedent conditions when only small volumetric portion of precipitation is transformed into streamflow. Above this threshold, event runoff coefficients are higher and increase rapidly with increasing soil moisture. This corresponds to wet antecedent conditions when a substantial volumetric portion of precipitation is transformed into streamflow. The original classification[6] additionally characterizes spatial and temporal properties of precipitation events and antecedent catchment conditions that are not used in this study due to a limited number of flood events compared with the number of all identifiable streamflow events of various magnitudes that were considered in the original study.

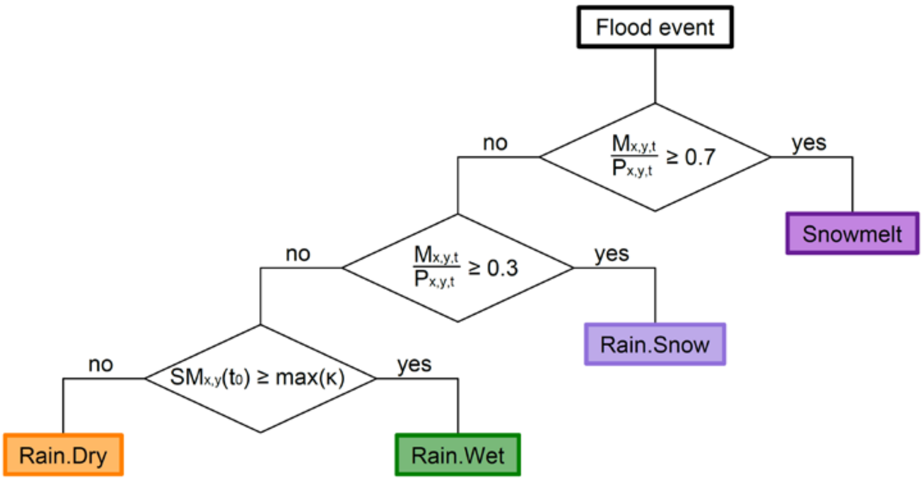

Supplementary Figure 7. A decision tree for a causative classification of flood events. $M_{x,y,t}$ is catchment- and event-averaged volume of snowmelt simulated by mHM in the total volume of flood-inducing precipitation event ($P_{x,y,t}$). $SM_{x,y}(t_0)$ is antecedent soil moisture simulated by mHM one day prior the start of the flood event. max(κ) is the point of maximum curvature of the fitted exponential function that describes a non-linear behaviour of runoff event coefficients and antecedent soil moisture for all runoff events identified by an the automated event separation
procedure[1].

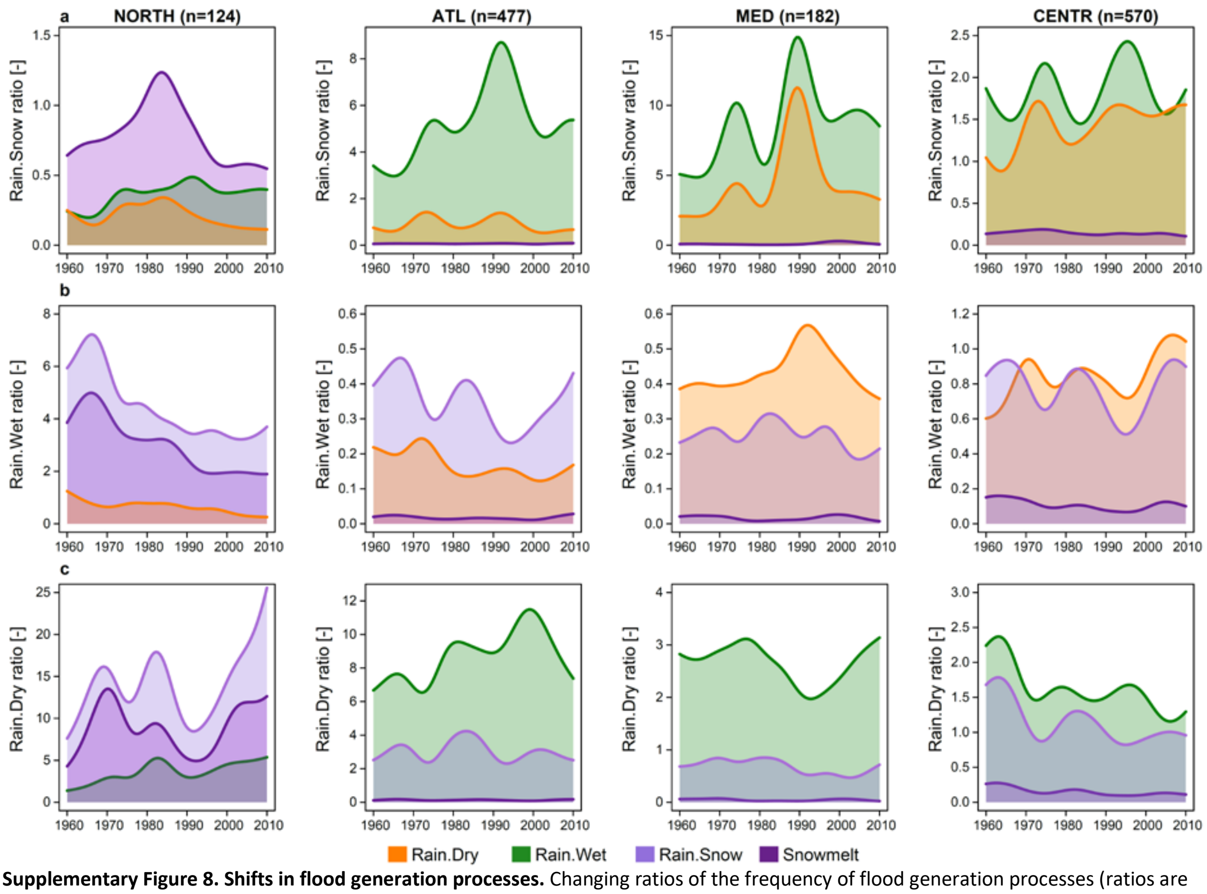

Supplementary Figure 8. Shifts in flood generation processes. Changing ratios of the frequency of flood generation processes (ratios are smoothed using kernel with bandwidth of 10 years) across four study regions with respect to **a** the frequency of Rain.Snow floods; **b** the frequency of Rain.Wet floods and **c** the frequency of Rain.Dry floods.

Supplementary Table 1. Significance of differences of flood magnitudes among four flood generation processes[1].

	NORTH				ATL				MED				CENTR			
	Rain. Dry n=470[2]	Rain. Wet n=923	Rain. Snow n=2910	Snow-melt n=1804	Rain. Dry n=2493	Rain. Wet n=15453	Rain. Snow n=4811	Snow-melt n=242	Rain. Dry n=2028	Rain. Wet n=4782	Rain. Snow n=1185	Snow-melt n=84	Rain. Dry n=8327	Rain. Wet n=11047	Rain. Snow n=7190	Snow-melt n=984
Rain. Dry																
Rain. Wet	p<0.01				p<0.01				p<0.01				p<0.01			
Rain. Snow	p<0.01	p<0.01			p<0.01	p=0.11			p<0.01	p<0.01			p<0.01	p<0.01		
Snow-melt	p<0.01	p<0.01	p=0.24		p<0.01	p=0.27	p=0.35		p=0.07	p<0.01	p<0.01		p<0.01	p<0.01	p<0.01	

[1] pairwise two-sided Kolmogorov-Smirnov test with false discovery rate correction

[2] n is the number of flood events generated by the corresponding process in the regional sample

Supplementary Note 5. Sensitivity analysis of regionally prevailing flood-rich and flood poor anomalies

To examine the effect of the exact definition of regionally prevailing flood-rich and flood-poor anomalies (Figure 4a and the Methods Section) on the significance of differences in the corresponding frequency of flood generation processes (Figure 4b) we repeat the Chi-squared test with false discovery rate correction among regions while varying the definition of the time windows by moving the start and end year of the window by one year at a time. Supplementary Table 2 shows that moving the start or end dates of the time windows by ±3 years does not significantly affect the results of the test, in most cases resulting in the exact same level of significance in the differences of flood generation processes as for the time windows displayed in Figure 4b. This means that regardless the selected exact starting and ending points of the regionally prevailing flood-rich and

flood-poor periods the proportions of flood generation processes are significantly different during anomaly periods compared to the whole study period.

Supplementary Table 2. The sensitivity of definition of regionally prevailing flood-rich and flood-poor anomalies (Figure 4) on the significance of differences[1] in the corresponding frequency of flood generation processes

	NORTH (n=124)		ATL (n=477)		MED (n=182)		CENTR (n=570)	
Periods as in Figure 4	p<0.01 (**)	p<0.01 (**)	p<0.01 (**)	p<0.01 (**)	p=0.02 (**)	p=0.02 (**)	p<0.01 (**)	p<0.01 (**)
**Starting year**								
-3 years	p<0.01 (**)	NA[2]	p<0.01 (**)	p<0.01 (**)	p=0.16	NA	p=0.36	p<0.01 (**)
-2 years	p<0.01 (**)	p<0.01 (**)	p<0.01 (**)	p<0.01 (**)	p=0.11	NA	p<0.01 (**)	p<0.01 (**)
-1 year	p<0.01 (**)	p<0.01 (**)	p<0.01 (**)	p<0.01 (**)	p=0.11	NA	p=0.02 (**)	p<0.01 (**)
+1 year	p<0.01 (**)	p<0.01 (**)	p<0.01 (**)	p<0.01 (**)	p<0.01 (**)	p=0.08	p=0.04	p<0.01 (**)
+ 2 years	p<0.01 (**)	p<0.01 (**)	p<0.01 (**)	p<0.01 (**)	p<0.01 (**)	p=0.02 (**)	p<0.01 (**)	p<0.01 (**)
+ 3 years	p<0.01 (**)	p<0.01 (**)	p<0.01 (**)	p<0.01 (**)	p<0.01 (**)	p=0.03 (**)	p=0.49	p<0.01 (**)
**Ending year**								
-3 years	p=0.02 (**)	p<0.01 (**)	p<0.01 (**)	p<0.01 (**)	p<0.01 (**)	p=0.31	p<0.01 (**)	p<0.01 (**)
-2 years	p<0.01 (**)	p<0.01 (**)	p<0.01 (**)	p<0.01 (**)	p<0.01 (**)	p=0.07	p<0.01 (**)	p<0.01 (**)
-1 year	p<0.01 (**)	p<0.01 (**)	p<0.01 (**)	p<0.01 (**)	p<0.01 (**)	p=0.02 (**)	p<0.01 (**)	p<0.01 (**)
+1 year	NA	p<0.01 (**)	p<0.01 (**)	p<0.01 (**)	NA	p=0.18	p=0.02 (**)	p<0.01 (**)
+2 years	NA	p<0.01 (**)	p<0.01 (**)	p<0.01 (**)	NA	p=0.10	p=0.27	p<0.01 (**)
+3 years	NA	p<0.01 (**)	p<0.01 (**)	p<0.01 (**)	NA	p=0.10	p=0.07	p<0.01 (**)

[1] Significance of these differences is evaluated using the Chi-squared test with false discovery rate correction (cases significant at the level α=0.05 of false discovery rate correction are indicated as ** in brackets after the exact p values of the individual Chi-squared test) for each regional flood anomaly. Red columns correspond to regionally prevailing flood-poor anomalies and blue columns correspond to regionally prevailing flood-rich anomalies (Figure 4a)

[2] NA indicates the cases when the shift of the corresponding time window is not possible due to the limits of the available time series (1960-2010)

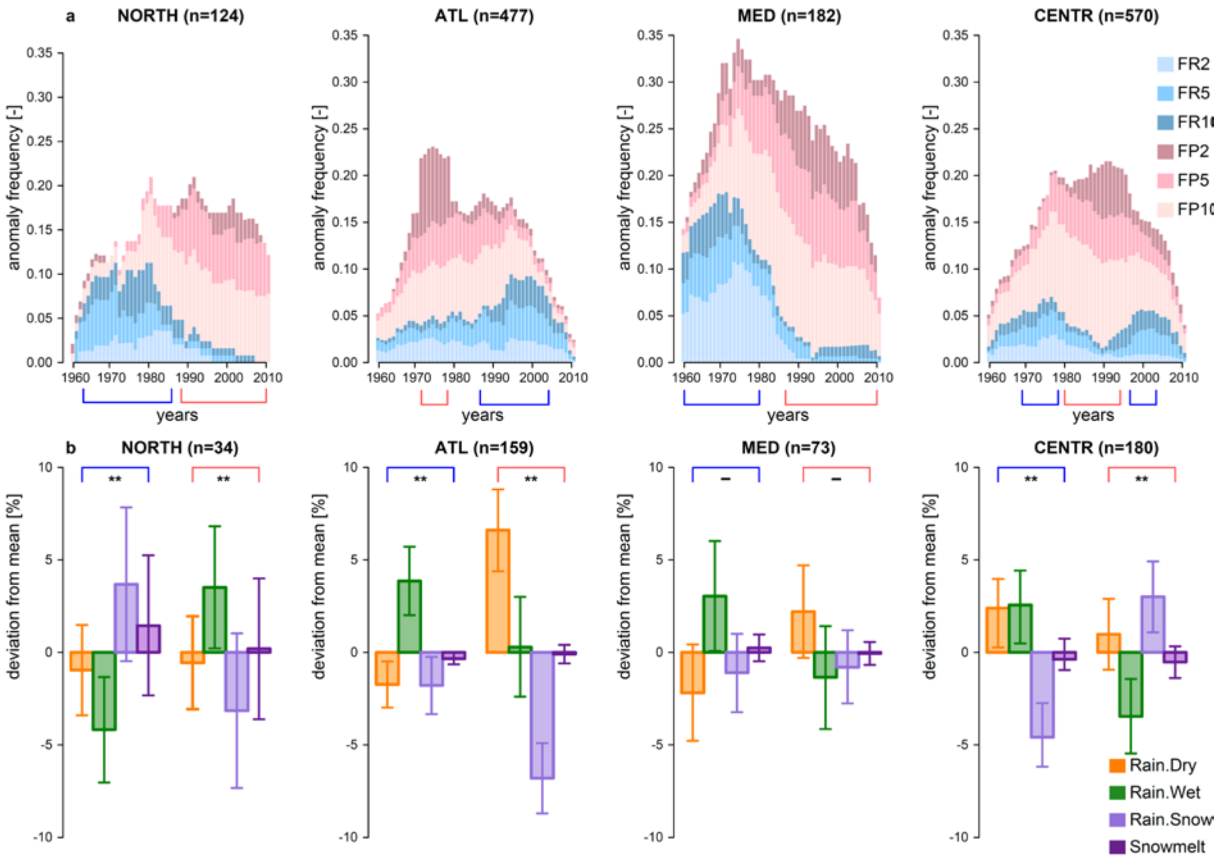

Supplementary Figure 9. Regional flood anomalies and shifts in flood generation processes. Same as Figure 4, but in **a** flood-poor anomalies are stacked on top of the flood-rich anomalies. **b** Display only catchments where at least one flood anomaly was detected. *n* is the number of considered catchments.

Supplementary Note 6. Split-sample analysis of probability distributions of flood magnitudes during two distinct time periods

We split the observational period into two periods (1960-1985 and 1985-2010) to analyse the significance of differences of the corresponding probability distributions of flood magnitudes for different flood generation processes using a pairwise two-sided Kolmogorov-Smirnov test with a correction for pairwise comparisons using the procedure based on the false discovery rate[7,8]. We compare the differences between different processes within the same period (Supplementary Table 3, rows and columns) and the differences in the probability distributions in the same process between different periods (Supplementary Table 3 diagonals; Supplementary Figure 10).

Supplementary Table 3. Significance of differences of flood magnitudes of four flood generation processes during two distinct time periods (1960-1985 and 1985-2010)[1].

	NORTH				ATL				MED				CENTR			
	Rain. Dry	Rain. Wet	Rain. Snow	Snow-melt	Rain. Dry	Rain. Wet	Rain. Snow	Snow-melt	Rain. Dry	Rain. Wet	Rain. Snow	Snow-melt	Rain. Dry	Rain. Wet	Rain. Snow	Snow-melt
Rain. Dry	p=0.02	p=0.04	p<0.01	p<0.01	p=0.25	p<0.01	p<0.01	p<0.01	p<0.01	p<0.01	p<0.01	p<0.01	p=0.65	p<0.01	p<0.01	p<0.01
Rain. Wet	p<0.01	p<0.01	p<0.01	p<0.01	p<0.01	p=0.25	p=0.05	p=0.21	p<0.01	p=0.03	p<0.01	p<0.01	p<0.01	p<0.01	p<0.01	p<0.01
Rain. Snow	p<0.01	p<0.01	p=0.64	p=0.32	p<0.01	p=0.01	<0.01	p=0.06	p<0.01	p=0.00	p=0.26	p<0.01	p<0.01	p<0.01	p<0.01	p=0.06
Snow-melt	p<0.01	p<0.01	p=0.58	p=0.91	p<0.01	p=0.05	0.01	p=0.03	p=0.67	p=0.27	p=0.67	p<0.01	p<0.01	p<0.01	p<0.01	p<0.01

[1] pairwise two-sided Kolmogorov-Smirnov test with false discovery rate correction. Significance of differences between probability distributions of flood magnitudes of different flood generation processes during the first period (1960-1985) displayed in columns (white background), significance of differences between two periods for the same flood generation process displayed on the diagonal (dark grey background), significance of differences of differences between different flood generation processes during the second period (1985-2010) displayed in rows (light grey background). The number of flood events in each sample is indicated in the Supplementary Figure 10.

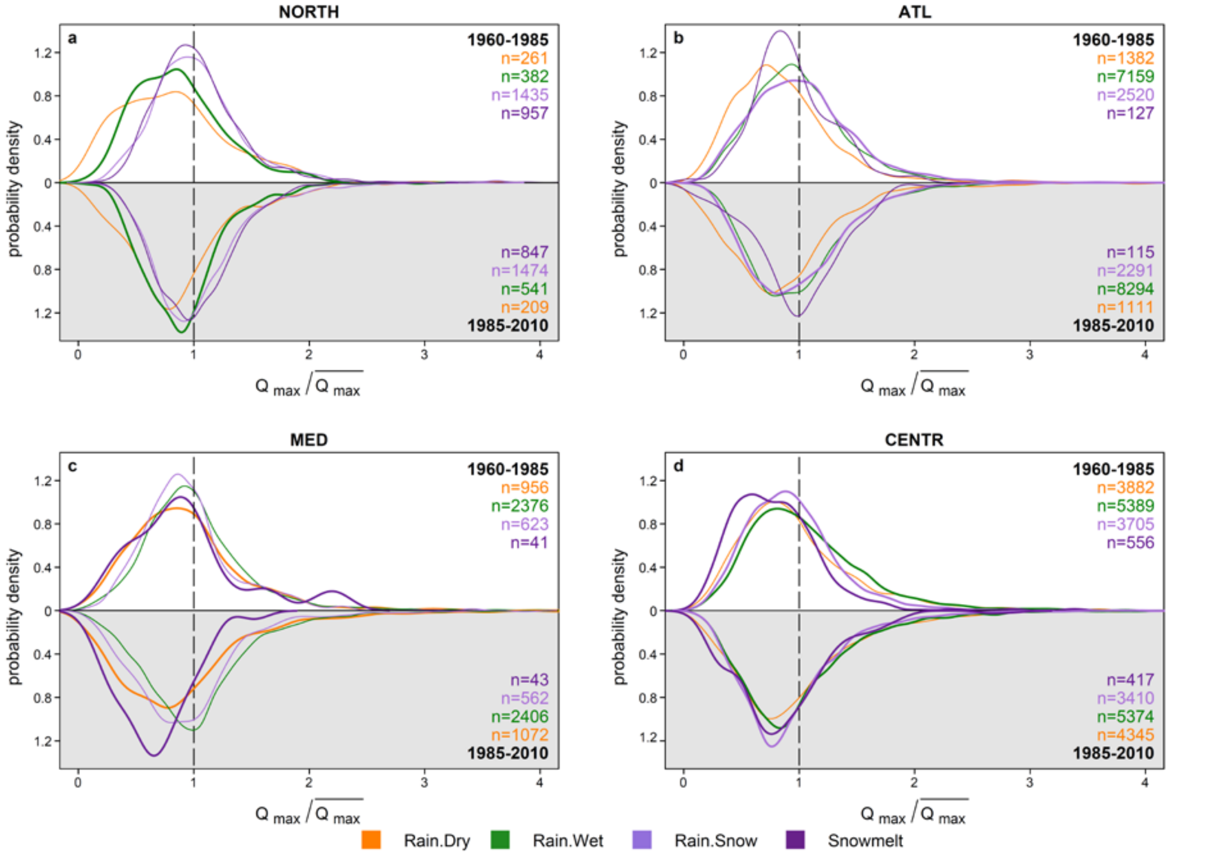

Supplementary Figure 10. Comparison of regional flood probability distributions of flood generation processes aggregated for two different periods. a-d Regional kernel density estimates of flood magnitudes (Qmax scaled by mean maximum annual flood for each study catchment) aggregated for each region and flood generation process. The right tails of probability density functions are only shown up to the value of 4 (four times the mean maximum annual floods). The significance of differences of flood magnitude distributions of flood generation processes aggregated for two different periods (1960-1985 (white background) and 1985-2010 (grey background)) is tested with the pairwise two-sided Kolmogorov-Smirnov test (α=0.01) applying a false discovery rate correction. Significant cases are displayed as thick lines. n is the number of flood events in each sample.

Supplementary Note 7. Regional probability distributions of precipitation properties for each flood generation process

Regional probability distributions of event precipitation volumes (sum of rainfall and snowmelt) and maximum precipitation intensities of four flood generation processes are estimated by scaling the series of these properties that correspond to annual flood peaks by their corresponding mean values in each catchment and pooling all catchments in a region, stratified by flood generation processes, and applying a kernel density estimator (Supplementary Figure 11 and Supplementary Figure 12). Additionally, we derived regional exceedance probability distributions and display them as semi-logarithmic plots to highlight the behaviour of the right tails of distributions.

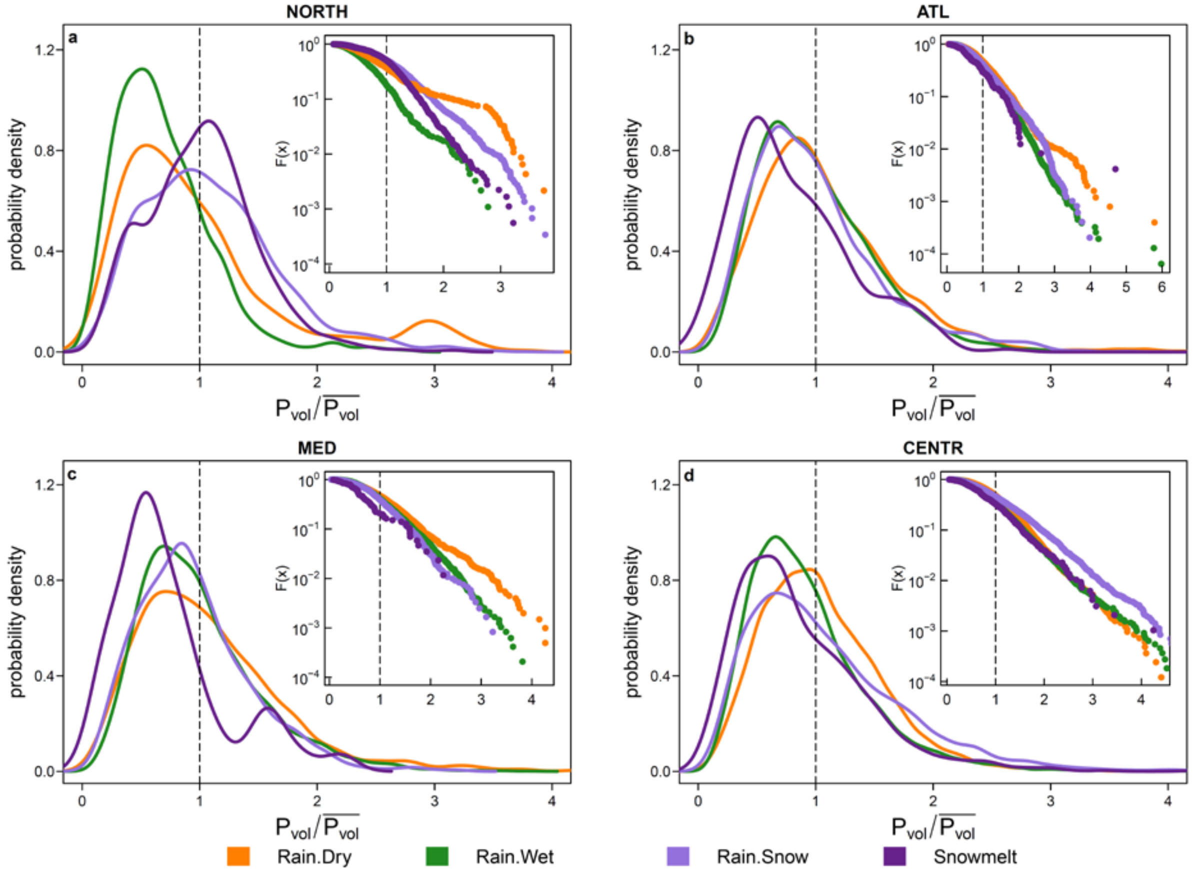

Supplementary Figure 11. Regional probability distributions of precipitation volumes that correspond annual flood maxima of different flood generation processes. a-d Kernel density estimates of precipitation volumes of annual flood maxima Pvol scaled by mean value of Pvol for all annual flood maxima of all flood events for each study catchment) aggregated for each region and flood generation process. The right tails of probability density functions are only shown up to the value of 4 (four times the mean value) and displayed in full in the corresponding insets. In all panels, the value of 1 on x-axes corresponds to the mean value of Pvol for all annual maximum floods. **Insets:** Semi-log plots of the exceedance probability of precipitation volumes associated with annual maximum floods (F(x), calculated using Weibull plotting position of each event individually for each generation process) for all study catchments visualizing particularly the right tails of regional distributions. The extent of x axes depends on the range of the recorded precipitation volumes in the catchments of corresponding region.

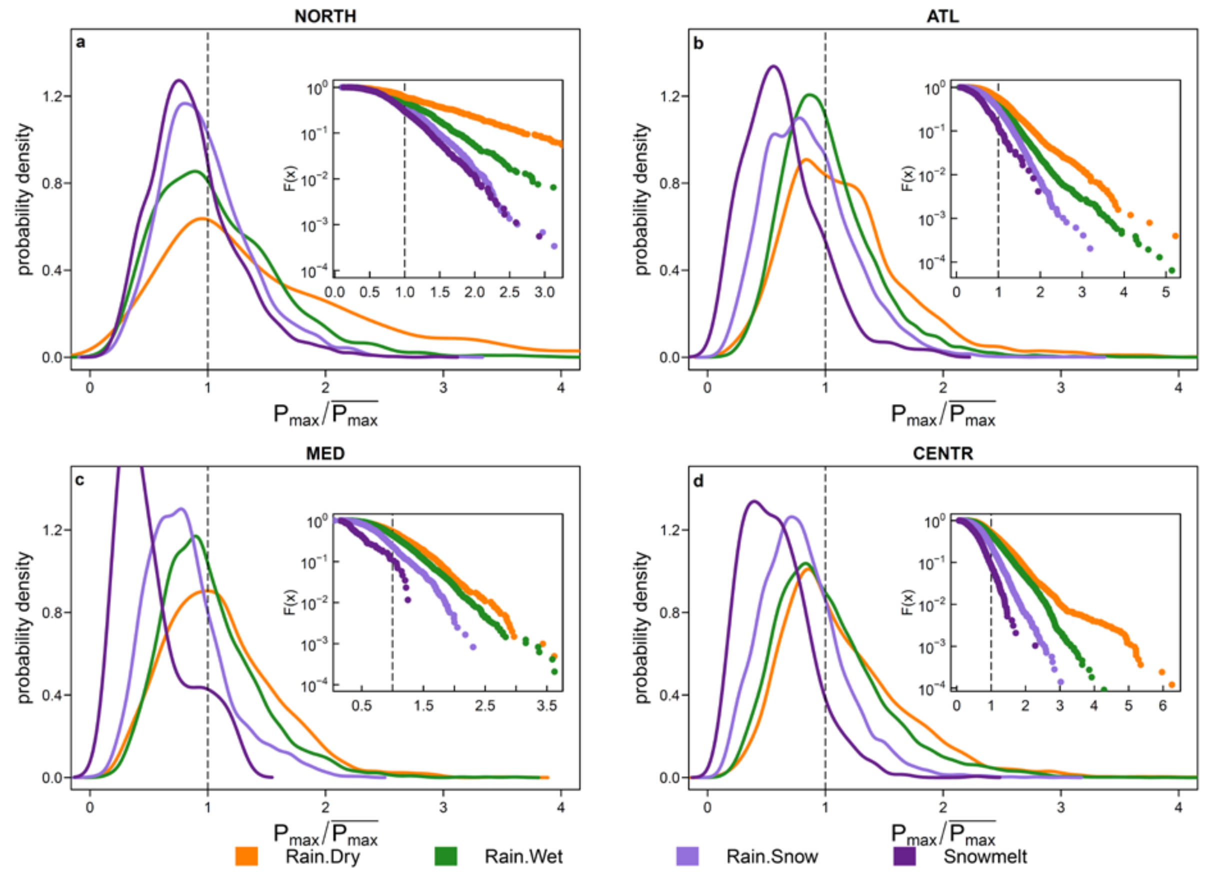

Supplementary Figure 12. Regional probability distributions of maximum precipitation intensities that correspond annual flood maxima of different flood generation processes. a-d Kernel density estimates of precipitation intensities of annual flood maxima Pmax scaled by mean value of Pmax for all annual flood maxima of all flood events for each study catchment) aggregated for each region and flood generation process. The right tails of probability density functions are only shown up to the value of 4 (four times the mean value) and displayed in full in the corresponding insets. In all panels, the value of 1 on x-axes corresponds to the mean value of Pmax for all annual maximum floods. **Insets:** Semi-log plots of the exceedance probability of precipitation intensities associated with annual maximum floods (F(x), calculated using Weibull plotting position of each event individually for each generation process) for all study catchments visualizing particularly the right tails of regional distributions. The extent of x axes depends on the range of the recorded precipitation intensities in the catchments of corresponding region.

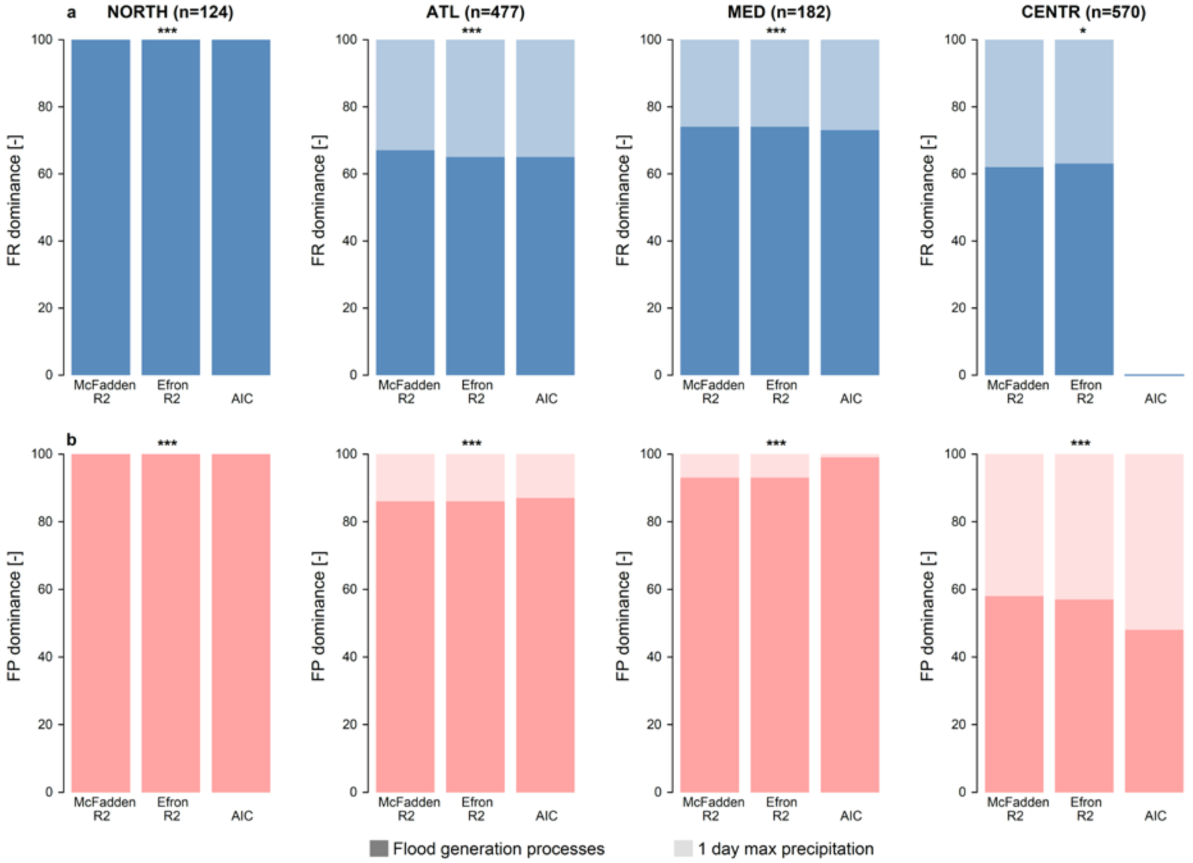

Supplementary Figure 13. General dominance (additional contributions) of temporal variations of flood generation processes and extreme precipitation for predicting the probability of occurrence of regional flood anomalies. Same as Figure 5, but for 1 day precipitation maxima.

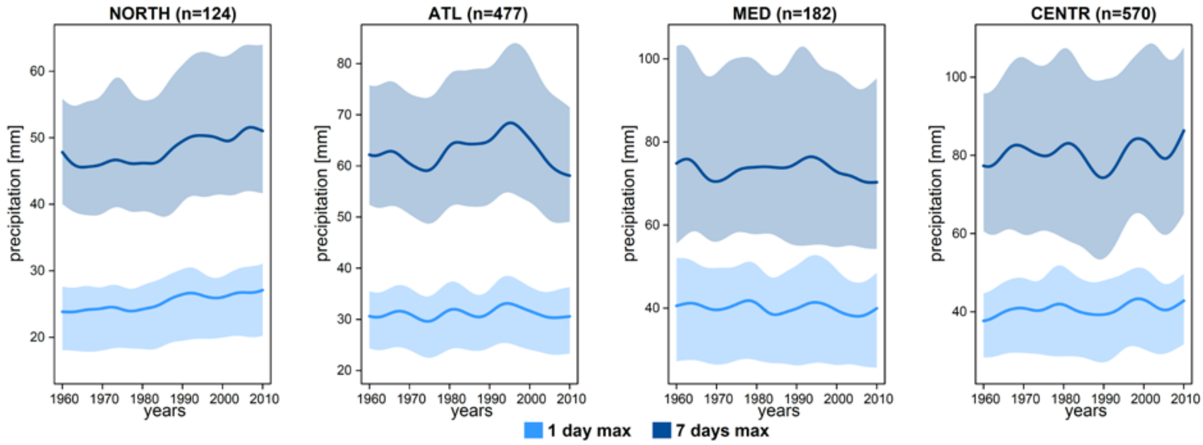

Supplementary Figure 14. Changes in precipitation maxima across European regions. Solid lines show the medians of 1 day precipitation maxima (light blue colour) and 7 days precipitation maxima for the period 1960-2010 (smoothed kernel with the bandwidth of 7 years) in the Northern (NORTH), Atlantic (ATL), Mediterranean (MED) and Central-Alpine (CENTR) regions. Shadow bands indicates spatial (i.e., inter-catchment) variability within the regions (25th and 75th percentiles). n is the number of catchments in the corresponding region.

Supplementary References

1. Tarasova, L., Basso, S., Zink, M., & Merz, R. Exploring Controls on Rainfall-Runoff Events: 1. Time Series-Based Event Separation and Temporal Dynamics of Event Runoff Response in Germany. *Water Resources Research*, *54*(10), 7711–7732; https://doi.org/10.1029/2018WR022587 (2018).
2. Institute of Hydrology. *Low Flow Studies*. (Wallingford, 1980).
3. Mei, Y., & Anagnostou, E. N. A hydrograph separation method based on information from rainfall and runoff records. *Journal of Hydrology*, *523*, 636–649; https://doi.org/10.1016/j.jhydrol.2015.01.083 (2015).
4. Merz, R., Blöschl, G., & Parajka, J. Spatio-temporal variability of event runoff coefficients. *Journal of Hydrology*, *331*(3–4), 591–604; https://doi.org/10.1016/j.jhydrol.2006.06.008 (2006).
5. Berghuijs, W. R., Woods, R. A., & Hrachowitz, M. A precipitation shift from snow towards rain leads to a decrease in streamflow. *Nature Climate Change*, *4*(7), 583–586; https://doi.org/10.1038/nclimate2246 (2014).
6. Tarasova, L. et al. A Process-Based Framework to Characterize and Classify Runoff Events: The Event Typology of Germany. *Water Resources Research*, *56*(5), 1–24; https://doi.org/10.1029/2019WR026951 (2020).
7. Benjamini, Y., & Hochberg, Y. Controlling the false discovery rate: a practical and powerful approach to multiple testing. *Journal of the Royal statistical society: series B (Methodological*), 57(1), 289-300 (1995).
8. Wilks, D. S. On “field significance” and the false discovery rate*. Journal of applied meteorology and climatology*, 45(9), 1181-1189 (2006).
